# Combination of Virtual Screening Protocol by *in Silico* toward the Discovery of Novel 4-Hydroxyphenylpyruvate Dioxygenase Inhibitors

**DOI:** 10.3389/fchem.2018.00014

**Published:** 2018-02-06

**Authors:** Ying Fu, Yi-Na Sun, Ke-Han Yi, Ming-Qiang Li, Hai-Feng Cao, Jia-Zhong Li, Fei Ye

**Affiliations:** ^1^Department of Applied Chemistry, College of Science, Northeast Agricultural University, Harbin, China; ^2^School of Pharmacy, Lanzhou University, Lanzhou, China

**Keywords:** HPPD inhibitors, structure-based design, pharmacophore model, molecule docking, virtual screening

## Abstract

4-Hydroxyphenylpyruvate dioxygenase (EC 1.13.11.27, HPPD) is a potent new bleaching herbicide target. Therefore, *in silico* structure-based virtual screening was performed in order to speed up the identification of promising HPPD inhibitors. In this study, an integrated virtual screening protocol by combining 3D-pharmacophore model, molecular docking and molecular dynamics (MD) simulation was established to find novel HPPD inhibitors from four commercial databases. 3D-pharmacophore Hypo1 model was applied to efficiently narrow potential hits. The hit compounds were subsequently submitted to molecular docking studies, showing four compounds as potent inhibitor with the mechanism of the Fe(II) coordination and interaction with Phe360, Phe403, and Phe398. MD result demonstrated that nonpolar term of compound 3881 made great contributions to binding affinities. It showed an IC_50_ being 2.49 μM against *At*HPPD *in vitro*. The results provided useful information for developing novel HPPD inhibitors, leading to further understanding of the interaction mechanism of HPPD inhibitors.

## Introduction

The success probability for novel herbicides discovery in agricultural field is cutting down and weeds are becoming widely resistant to most common used herbicide in recent years (Green, 2014), all these make weed management difficult and time consuming. According to the literature, at least 315 weed biotypes, 183 weed species including 110 dicots and 73 monocots worldwide have been reported to have acquired resistance to widely used herbicides (Vishnoi et al., 2009). Thus, there is an urgent need for novel herbicide discovery to overcome the weeds resistance. The 4-hydroxyphenylpyruvate dioxygenase (HPPD) inhibitor offers such solutions by bleaching herbicide mode of action.

HPPD, which was founded by Zeneca Group PLC in 1982, is nonheme Fe(II)-containing dioxygenases (Lee et al., 1997; Neidig et al., 2006). HPPD catalyzes the conversion of *p*-hydroxyphenylpyruvate (HPPA) to homogentisate (HGA) in aerobic metabolism. This reaction involves decarboxylation, substituent migration and aromatic oxygenation in a single catalytic cycle (Moran, 2005; Purpero and Moran, 2006). The transformation catalyzed by HPPD has both agricultural and therapeutic significance. In plants, HPPD is a key enzyme involving the biosynthesis of the prenylquinones, plastoquinone and tocopherols, and has been used for selective weed control since the early 1990s (Wu et al., 2002). Tocopherol and plastoquinone are produced by further transformation of HGA, and both of them are vital for the natural growth of plants (Yang et al., 2004). In the carotenoid biosynthesis pathway, plastoquinone is an essential cofactor for phytoene desaturase. HPPD inhibition will hinder HPPA–HGA conversion, which gives rise to the deficiency in isoprenoid redox cofactors such as plastoquinone and tocopherol, and finally causes growth inhibition, necrosis and death of treated plants (Borowski et al., 2004; Kovaleva and Lipscomb, 2008; Siehl et al., 2014). In human, the deficiency of tyrosine catabolism enzyme will lead to the tyrosine catabolism pathway suffocation (Raspail et al., 2011).

**Figure 1:**
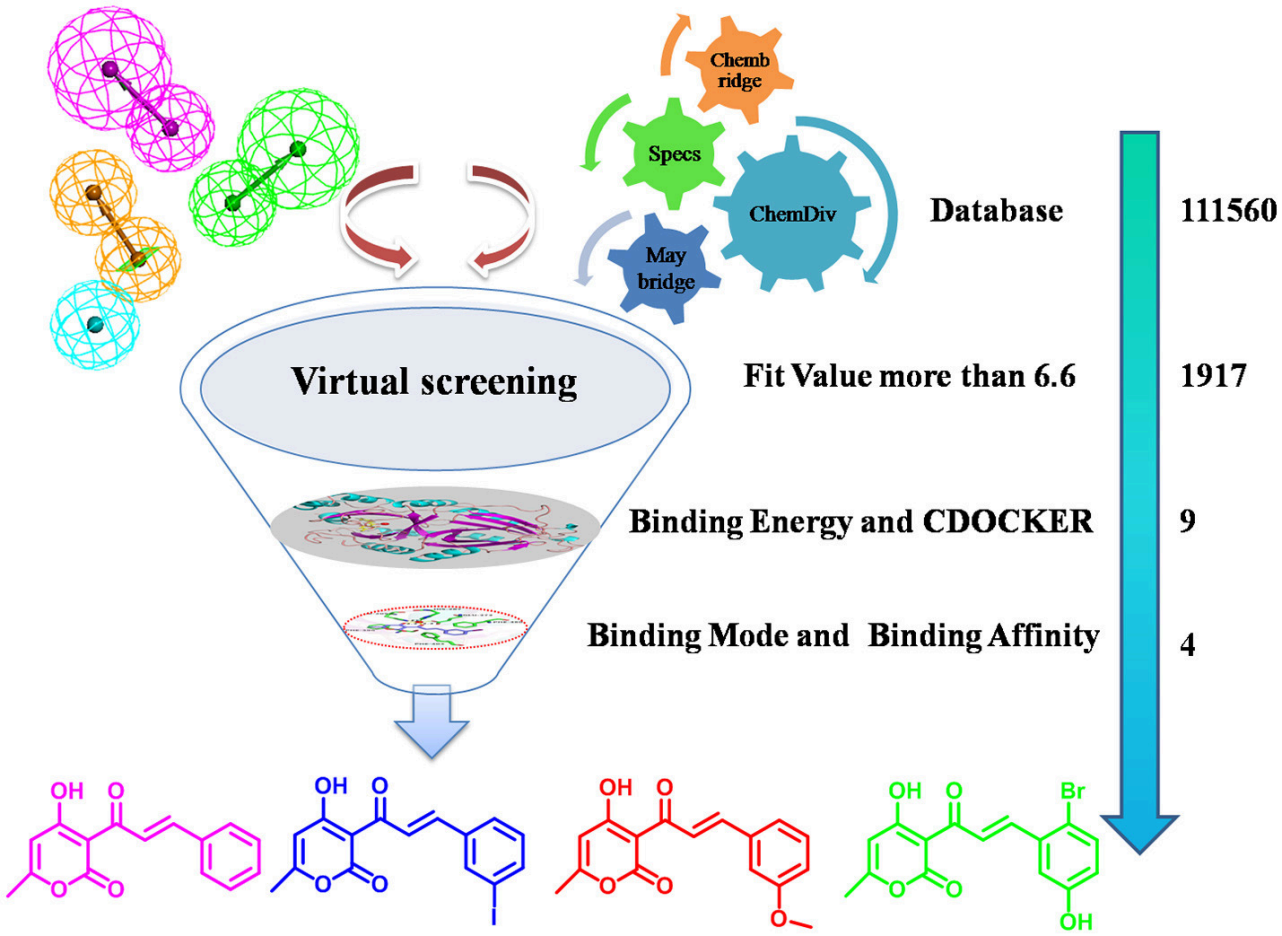
The screening workflow that was applied to discern novel HPPD inhibitors.

HPPD is a kind of new herbicide target enzyme, and several HPPD inhibited herbicides have been commercialized. Since the first launch of pyrazolynate by Sankyo in 1980, approximately 13 HPPD inhibitors have been commercialized (Meazzaa et al., 2002; Witschel, 2009). Several of class HPPD inhibitors are currently used as selective broad leaf herbicides including triketones, pyrazoles, isoxazoles, diketone nitriles and benzophenones, among them, the triketone herbicides have contributed to various commercialized HPPD inhibitors through structural modification (Figure S1), such as sulcotrione, mesotrione and benzobicylon (Mitchell et al., 2001; Sutton et al., 2002; Ahrens et al., 2013). These inhibitors also show numerous advantages, such as application security, high activity, low residual, broad-spectrum weeds control (including herbicide-resistant weed biotypes), excellent crop selectivity and benign environmental effects (Beaudegnies et al., 2009; Woodyard et al., 2009). Many have been obtained potent inhibitors but none was as potent as sulcotrione. Because of emergence of sulcotrione resistant, therefore obtaining HPPD inhibitors with novel scaffolds is an urgent task for herbicide developers. The inhibitory mechanism has been found in previous studies (Lin et al., 2013; Silva et al., 2015). In HPPD, active site Fe(II) octahedral coordination sphere is accomplished by forming coordinating interaction with three protein ligand atoms and three water molecules. However, two coordinating water molecules are displaced by the 1,3-diketone moiety of the HPPD inhibitor in the enzyme-inhibitor complex. HPPD enzyme shares two His and a Glu residue as Fe(II) ligands and the distance to oxygen atom of His is 2.5 Å (Brownlee et al., 2004). Two other bidentate ligands are from the oxygen of HPPD inhibitors and the distance from oxygen to metal ions is between 1.9 and 2.4. The π-π stacking interaction also plays an important role in the binding model of complex (Ndikuryayo et al., 2017). Based on the above research, a virtual screening method was used to obtain novel HPPD inhibitor. In the present investigation, predictive 3D-pharmacophore model was built on basis of the known HPPD inhibitors reflecting the structure-activity relationship (SAR). Subsequently, the best pharmacophore model was used as a 3D query for searching four databases (Maybridge, Chembridge, ChemDiv, and Specs) to discern novel HPPD inhibitors and also utilized as a predictive program to estimate bioactivity of HPPD inhibitors. Further, molecular docking and molecular dynamics (MD) simulation were performed to identify the most potential HPPD inhibitors with strong ligand binding affinity. The obtained compounds were subsequently assayed the bioactivity *in vitro* to verify the inhibition on *At*HPPD. The detailed screening workflow was shown in Figure 1.

## Materials and methods

### Data collection and preparation

**Figure 2:**
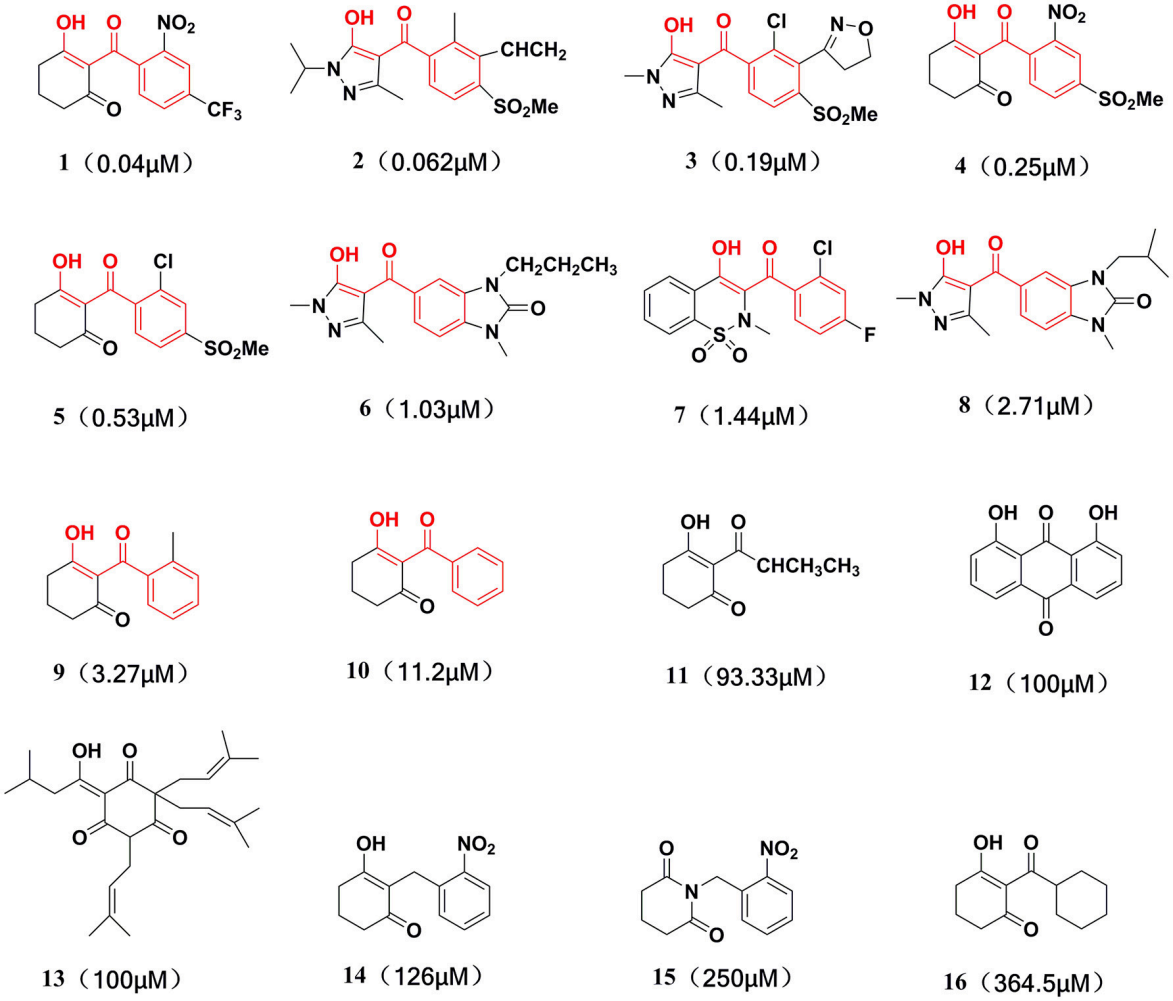
Training set compounds with IC_50_ values used for pharmacophore model generation.

**Figure 3:**
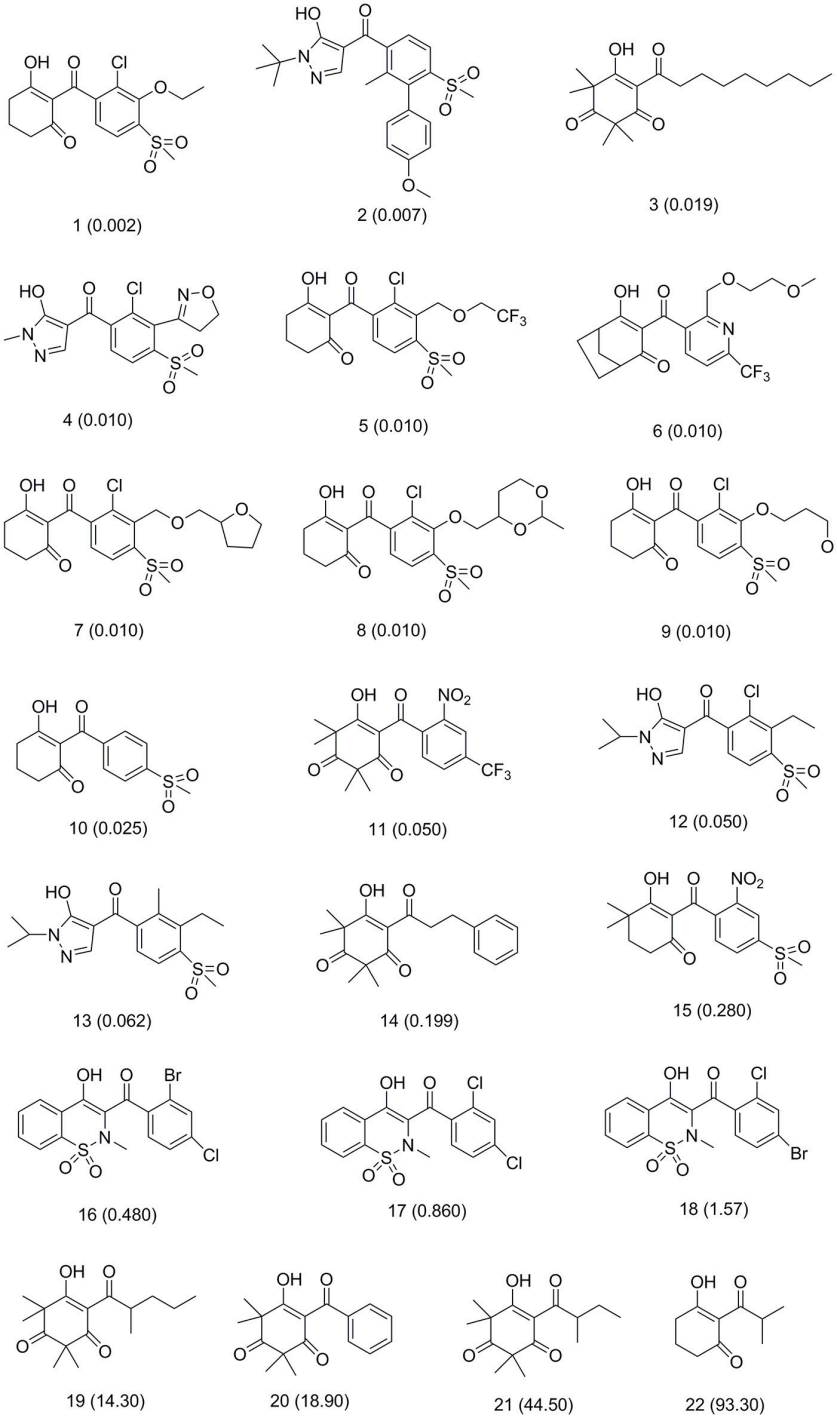
Test set compounds with IC_50_ values used for pharmacophore model validation.

This training set, including 16 structurally diverse chemical compounds (Figure 2) from various literatures (Huang et al., 2002; Cho et al., 2013; Xu et al., 2014; Lei et al., 2016) with wide activity were used to generate 3D-pharmacophore models. The molecular structures of the studied dataset compounds were depicted in SYBYL 6.9 program (SYBYL, Version 6.9^1^). To validate the hypothesis, the test set 22 compounds obtained from literatures (Huang et al., 2002; Dayan et al., 2009; Cho et al., 2013; Lei et al., 2016) were prepared using the same protocol (Figure 3).

### 3D-pharmacophore generation using HypoGen

Based on spatial permutations of pharmacodynamic characteristic elements that play an important role in the activity, the key common chemical features were selected to create 3D-pharmacophore models with HypoGen. The common features of pharmacophore were generated in “Feature Mapping” module by identifying the important chemical features from the training set before establishing hypothesis. Hydrogen bond acceptor (HBA), hydrogen bond donor (HBD), hydrophobic features (HY), and ring aromatic (RA) were selected as key chemical properties to generate the pharmacophore model. Subsequently, pharmacophore models were computed using the “HypoGen” module of DS v3.5 (Catalyst, Version 4.10^2^) and the top 10 hypotheses based on the fixed cost, null cost, and total cost values were saved. The best hypothesis (Hypo1) model was analyzed and selected according to the cost analysis and consideration of vital factors, namely configuration cost, root mean square deviation *(rmsd)* and correlation coefficient (*r*) of the training set between experimental and predicted values.

### Validation and evaluation of pharmacophore model

The best ligand-based pharmacophore model evidenced that as *rmsd* and configuration components are very low and the value of total cost would always be rather higher than null cost but close to the fixed cost. Regard to external validation, firstly the correlation of test set between the experimental and predicted activity was an important index. Secondly randomizing the data using Fischer's Randomization test was performed to obtain cross validation. All process was carried out with the “Ligand Pharmacophore Mapping” module.

### Pharmacophore based on database virtual screening

The well-validated Hypo1 was further used to screen the database consisted of 111560 compounds belonging to four different chemical databases. The four databases were built by “Build 3D Database” protocol of DS v3.5. Subsequently, Chembridge, Maybridge, ChemDiv and Specs were selected as “Input Database” and Hypo1 was imported to “Input Pharmacophore” to discriminate potential HPPD inhibitor from database using “Search 3D Database” of DS v3.5.

### Molecular docking

1917 Compounds which were obtained according to the property of fit value and predictive bioactivity in the previous steps further were used to molecular docking. Docking simulation was carried out utilizing CDOCKER module of the DS v3.5 to explore the binding mode of compounds. The *At*HPPD crystal structure (PDB ID: 1TFZ) was downloaded from the Protein Data Bank. The protein was prepared by removing the water, adding hydrogen and correcting the incomplete residues using “Clean Protein” tool in “Prepare Protein” module, then the protein were assigned potentials with CHARMm force field. The active site of protein was predicted and identified using “Edit binding site” module of DS v3.5 according to the native ligand and the radius was set to 10Å. The obtained receptor was used as the “Input Receptor” molecule parameter. All hit compounds subjected to first filtering processes were chosen as “Input Ligand” and docked into the active site of HPPD. The “Pose Cluster Radius” was defined as 0.5 Å for increasing the diversity of the docked poses. The Top Hits was set to 10, which means top the 10 conformations were saved for each ligand based on scoring and ranking by the negative value of CDOCKER energy. The remaining parameters were default. The best binding modes were determined by docking scores and also the comparison with available complex crystal structure of DAS869 with *At*HPPD as reference.

### Molecular dynamics simulations

In order to analyze the ligand-target interactions, further 9 hits compounds with the best docked poses in complex with *At*HPPD were submitted to the MD simulation in Amber16 (Case et al., 2017). Meanwhile, mesotrione and 2-(aryloxyacetyl)cyclohexane-1,3-diones were used as positive control for the *in silico* simulations. The general AMBER force field gaff and ff14SB force field were employed for the ligand and protein, respectively (Wang et al., 2004; Hornak et al., 2006). The 3D structure coordinate files of candidate ligand and control compound were manually edited to match atom number and naming conventions consistent with pdb format for input into the “Antechamber” module of Amber16. The partial atomic charges of ligands were calculated using the AM1-BCC method (Jakalian et al., 2002). The force field of the Fe(II) treated in the “MCPB” module of Amber which was used to build nonbonded model. The nonbonded model with simple form and excellent transferability implemented in the metal center parameter builder (MCPB) tool was employed to treat Fe(II)-protein interaction (Peters et al., 2010; Li and Merz, 2014). The side chain model including Fe(II) coordination sphere with His205, His287, and Glu373 was first created in MCPB module. The geometry optimization and the atomic partial charges of side chain model were calculation through the restrained electrostatic potential (RESP) technique in Gaussian03 (Frisch et al., 2004). Then parameter information included bond, angle, torsion, improper, van der Waals, and electrostatic terms about the Fe(II) coordinating three residues were generated in the “MCPB” module of Amber. The charge neutralized and solvated progress was performed in the “LEaP” module of Amber16. The resulting structure was immersed into a TIP3P water box with 11,614 water molecules in a rectangular periodic box of 10 Å distance around the complex and eight sodium counter ions were added to maintain electro-neutrality of all systems. The energy minimization and equilibration protocol was carried out in the Sander program. First, a minimization with a tightly restrained protein with a force constant of 500 kcal mol^−1^ Å^−2^ was applied to all the atoms of the complex to relieve bad contacts in the surrounding solvent. Then, only the protein backbone atoms were fixed with a restraint force of 5.0 kcal mol^−1^ Å^−2^ to minimize the side chain and ligand. Finally, a minimization was performed for all atoms with no restraint. In each step, energy minimization was first performed using the steepest descent algorithm for 2,500 steps, and then the conjugated gradient algorithm for another 2,500 steps. The temperature was gradually raised from 0 to 298 K in the canonical (NVT) ensemble with Langevin thermostat to relax the location of the solvent molecules. Then short equilibration with 500 ps was performed to adjust the solvent density in the isothermal isobaric (NPT) ensemble with Monte Carlo barostat. Finally the system was equilibrated for 1 ns without any restraint in the NTP ensemble. All simulations were run with the PMEMD program without any restraints for 10 ns in NTP ensemble (298 K and a pressure of 1 atm) with a 2 fs time step. During the MD simulation, the particle mesh Ewald (PME) algorithm was used to deal with long-range electrostatic interactions, with a cut-off distance of 10Å (Darden et al., 1993; Essmann et al., 1995). And bond lengths involving hydrogen atom were constrained using the SHAKE algorithm (Ryckaert et al., 1977).

The binding free energy (Δ*G*_bind_) was calculated with the molecular mechanics and Poisson–Boltzmann solvation area (MM/PBSA) methodology was applied based on stable MD trajectory. Entropy was omitted herein because we pay more attention to relative binding free energy for a series of very similar systems.

(1) $$\begin{array}{l} \Delta G_{binding}=G_{complex}-G_{receptor}-G_{ligand} \end{array}$$

Where *G*_*complex*_, *G*_*receptor*_, and *G*_*ligand*_ are the free energy of complex, receptor and ligand molecules, respectively. The free energy (*G*) was calculated based on an average over the extracted snapshots from the MD trajectories. Each term can be expressed as follows:

(2) $$\begin{array}{l} G=E_{gas}+G_{sol}-TS \end{array}$$

(3) $$\begin{array}{l} E_{gas}=E_{\mathrm{int}}+E_{vdw}+E_{ele} \end{array}$$

(4) $$\begin{array}{l} Gsol=G_{GB}+G_{np} \end{array}$$

(5) $$\begin{array}{l} G_{np}=\gamma SASA \end{array}$$

Where *E*_*gas*_ is gas-phase energy and can be decomposed into internal energy *E*_int_, van der Waals energies *E*_*vdw*_ and Coulomb *E*_*ele*_; *G*_*sol*_ is salvation free energy; *G*_*GB*_ is the polar solvation contribution calculated by solving the GB equation; *G*_*np*_ is the nonpolar solvation contribution and was estimated by the solvent accessible surface area (SASA) determined using a water probe radius of 1.4 Å (Yang et al., 2011). The surface tension constant (γ) was set to 0.0072 kcal mol^−1^ Å^−2^ (Sitkoff et al., 1994); *TS* is entropy term.

Free energy decomposition was performed to obtain the key residues contribution to the binding energy of inhibitors. MM-PBSA was used to compute the interaction energies to each residue in Amber16 by considering molecular mechanics energies and solvation energies without considering the contribution of entropies. The binding interaction between residue-ligand pair comprises three terms: the van der Waals contribution (Δ*E*_*vdw*_), the electrostatic contribution (Δ*E*_*ele*_) and the solvation contribution (Δ*G*_*sol*_).

(6) $$\begin{array}{l} \Delta G_{inhibitor\text{\_}residue}=\Delta E_{vdw}+\Delta E_{ele}+\Delta G_{sol} \end{array}$$

### *At*HPPD inhibition bioassay *in Vitro*

Recombinant *At*HPPD and homogentisate 1,2-dioxygenase (HGD) was derived from Guangfu Yang research team. HPPD inhibitory activity was determined by using method of coupled enzyme in previously published works (Wang et al., 2015). Assays were performed in 96-well plates at 30°*C* using a UV/vis plate reader to monitor the generation of maleylacetoacetate at 318 nm. The IC_50_ was then calculated based on the plot of the residue activity against different concentration of test compounds at certain concentrations of substrate by fitting the curves. Each experiment was three replicates and averaged. Compound 3881 was purchased from Innochem. Mesotrione was obtained from Wuhan Yuancheng Technology Co. Ltd. and was recrystallized before use.

## Results and discussion

### HypoGen model generation for HPPD inhibitors

**Figure 4:**
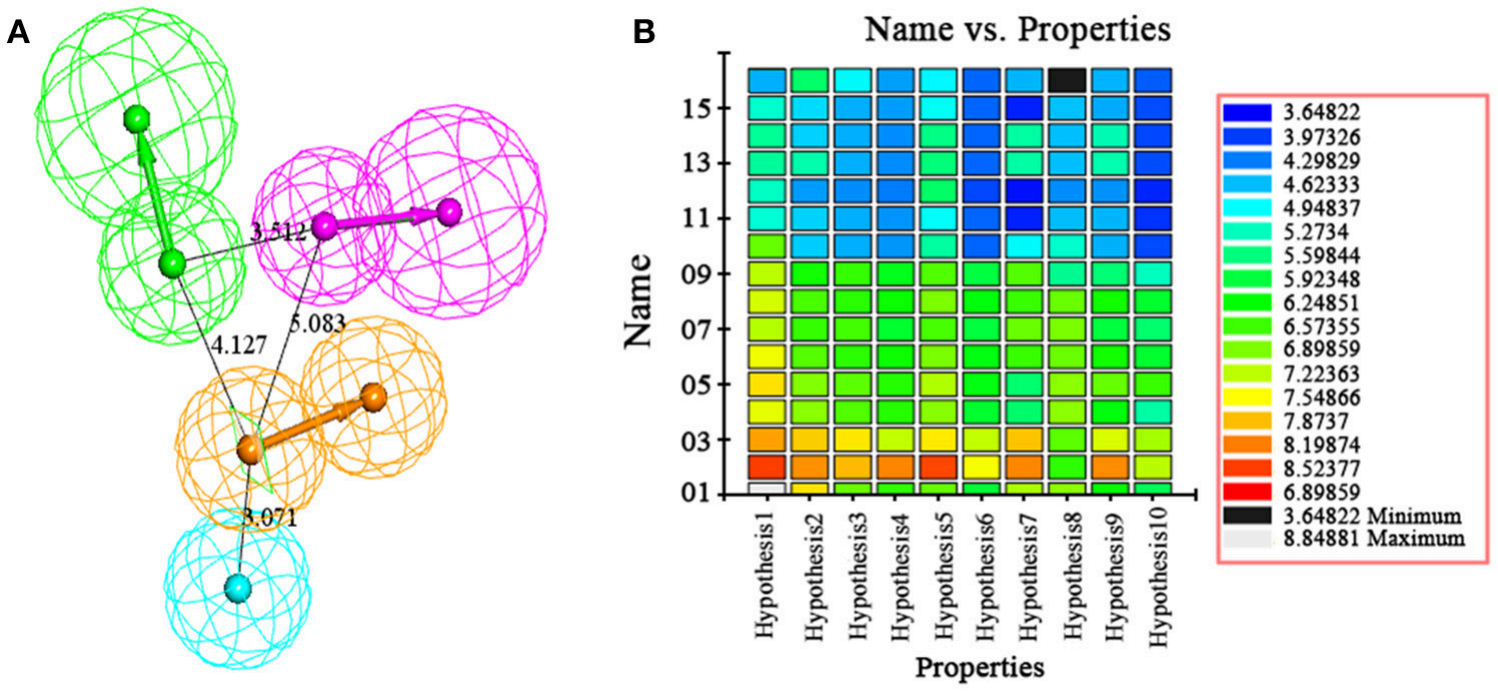
**(A)** The best pharmacophore, Hypo1, was shown with its inter-feature distance constraints in angstrom (Å), where HA, HBD, RA, and HBA were illustrated in cyan, pink, orange, and green, respectively. **(B)** Heat map of the ten hypotheses from training set.

The best quantitative pharmacophore models indicated that HBA, HBD, HA and RA features were important. The top 10 pharmacophore hypotheses were generated based on the structure and the activity values of the training set compounds. The values of 10 hypotheses such as total cost, *r*, and *rmsd* were statistically significant (Table 1). All model total cost was from 72.491 to 94.376. The configuration cost of 16.09 bits represented the acceptable complexity of the hypotheses space. The fixed and the null cost values were 63.670 and 195.34, respectively. Hypo1 had best *r* (0.978), maximum fitvalue (10.559) and lowest *rmsd* of 0.907. The total cost (72.491) was also low and closed to the fixed cost (63.670). Also, the cost difference 122.85 between null and fixed cost was more than 70 bits, which illustrated that total cost was far from the null cost. Comparing with other hypotheses, Hypo1 with higher ΔCost, better *r* and low *rmsd* accounted for all the pharmacophore features and had good predictive ability. Therefore, Hypo1 selected as a best hypothesis was used for further analyses. The Hypo1 chemical features with its geometric parameters were shown in Figure 4A and matched heat map of the ten hypotheses from training set was displayed in Figure 4B. The results indicated that all the training compounds were well matched to the Hypo1 and the compounds with better activity and fitvalue tended to red.

**Table 1:** Parameters of top pharmacophore hypotheses computed by *HypoGen* algorithm^a^.

**Hypo No**.	**Total cost**	***rmsd***	***r***	***^a^*Δcost**	**Features**	**Maximum fit**
Hypo 1	72.49	0.91	0.98	122.85	HBA,HBD,HY,RA	10.56
Hypo 2	82.96	1.52	0.94	112.38	HBA,HY,HY,RA	9.57
Hypo 3	84.73	1.61	0.93	110.61	HBA,HY,HY,RA	9.12
Hypo 4	85.02	1.62	0.93	110.32	HBA,HY,HY,RA	8.94
Hypo 5	89.14	1.74	0.92	106.20	HBA,HBD,HY,RA	9.97
Hypo 6	90.97	1.85	0.90	104.37	HBA,HBA,HY,HY	8.36
Hypo 7	92.52	1.90	0.90	102.82	HBA,HY,HY,HY,RA	9.55
Hypo 8	93.52	1.91	0.90	101.82	HBA,HBD,RA	6.98
Hypo 9	93.93	1.93	0.90	101.41	HBA,HBD,HY,RA	9.27
Hypo10	94.38	1.96	0.89	100.96	HBA,HBA,HY,HY	8.13

a Δ*Cost = null cost–total cost, null cost = 195.34, fixed cost = 63.67, configuration = 16.09*.

### Pharmacophore model Hypo1 validation

Verification hypothesis is one of the important processes in the formation of pharmacophore. Test set and Fisher's Randomization test were employed to confirm the quality of pharmacophore.

**Figure 5:**
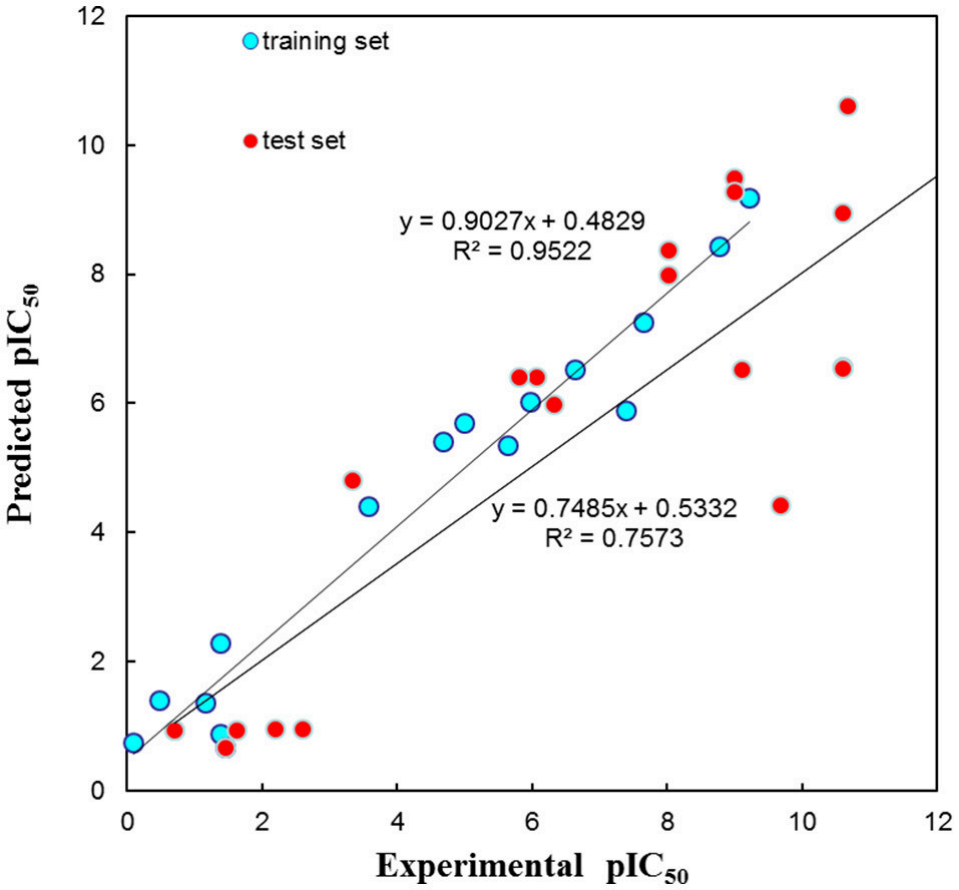
The graph of experimental vs. predicted activities by Hypo 1.

The test set was used to validate whether the best hypothesis was capable of prediction for the active compounds other than the training set molecules (Debnath, 2002). Hypo1 has predicted the biological activities of most of the test set compounds in the same activity scale with the *r* being 0.757 (Figure 5). The *r* for the training set given by Hypo1 was 0.952. This result suggested that the Hypo1 was not only fit for training set compounds but also for the external compounds. It was also verified legalization of Hypo1 to screen databases.

**Figure 6:**
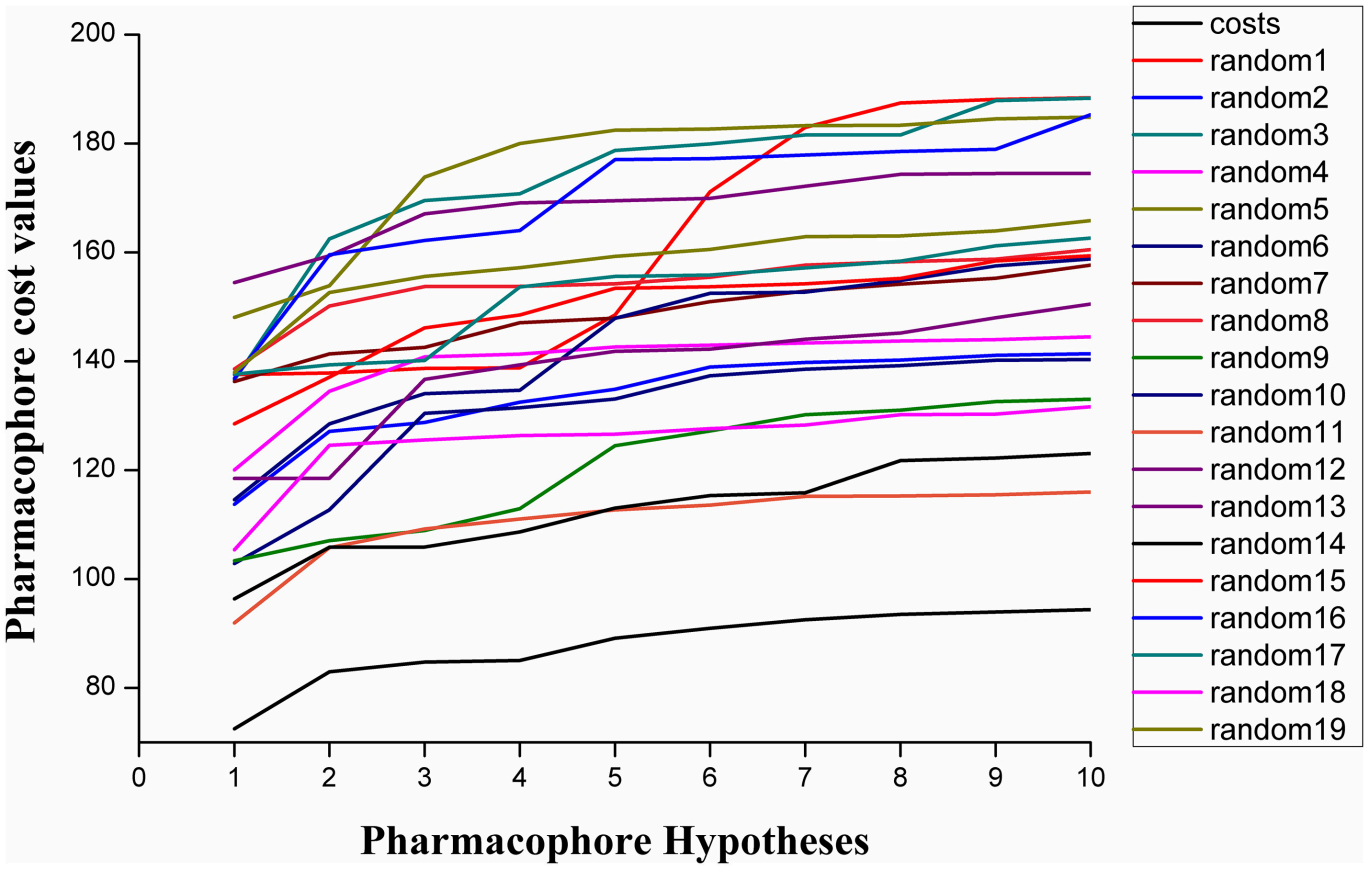
The difference in costs between HypoGen runs and the scrambled runs. The 95% confidence level was selected.

**Figure 7:**
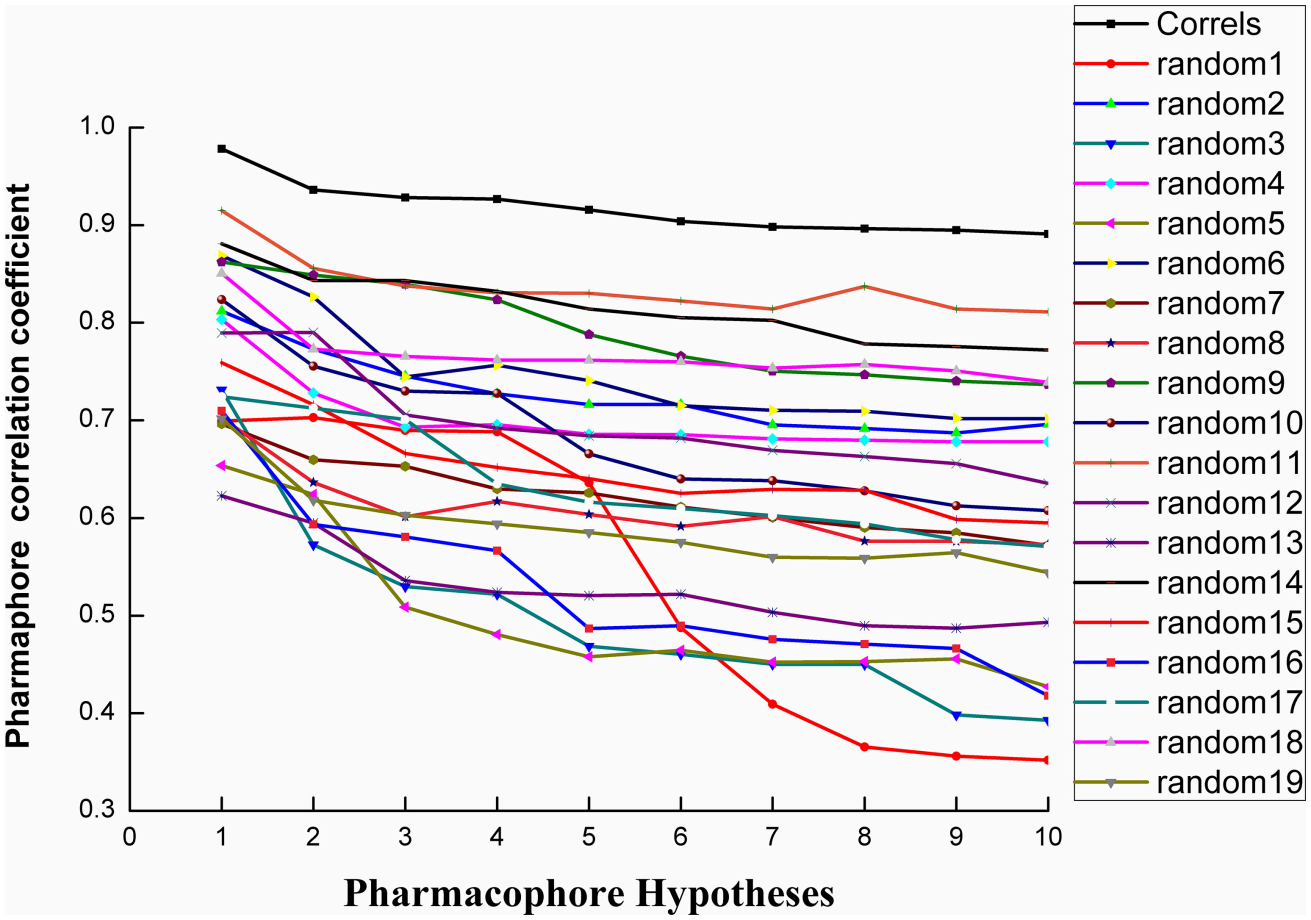
The difference in correlation coefficient between HypoGen runs and the scrambled runs. The 95% confidence level was selected.

Fischer randomization calculations were performed on the training set to validate statistical robustness of Hypo1 (Sakkiah et al., 2010). 19 Random hypotheses were generated in 95% confidence level and compared with Hypo1, and it was found that the total cost of Hypo1 was lower than that of 19 random models (Figure 6), which meant that the Hypo1 was robust and stable. Meanwhile, statistical *r* of Hypo1 was far more superior to the randomly generated 19 hypotheses (Figure 7).This result clearly indicated that Hypo1 was not generated occasionally, and the relationship between the structures and bioactivity did exist in the training. Based on these validation results, the best validated Hypo1 as a 3D query was used to retrieve HPPD inhibitors with novel scaffold from four databases.

### Database screening analysis

Virtual screening based on chemical databases is a fast and accurate approach to identify novel and potential drug (Gogoi et al., 2016; Guedes et al., 2016). The Hypo1 have used as a 3D query tool to screen the chemical databases like Maybridge (20565), Chembridge (40637), ChemDiv (12620), and Specs (37738). According the average fitvalue of the training set was approximately 6.6 and the compounds with a value greater than 6.6 were screened from four databases. As a result, 643, 392, 416, and 466 (a total of 1917) compounds from Maybridge, Chembridge, ChemDiv and Specs, were mapped upon all the features of Hypo1. Screening compounds had scored the HypoGen estimated activity value between 0.01 and 7.3 μM, and thus considered best for further studies.

### Molecular docking analysis

**Figure 8:**
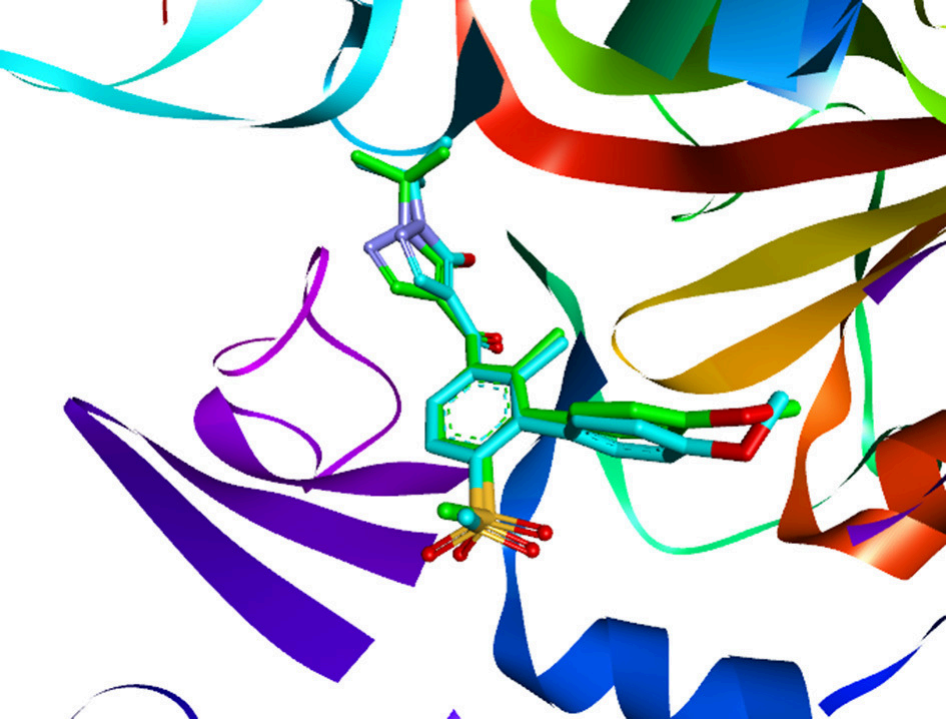
The ligand compared by the CDOCKER docking method. Redocked ligand was green and the native ligand in the crystallographic complex was cyan.

Molecular docking is a perfect method for predicting the interaction between small molecules and the receptor binding cavity at the molecular level. In the current study, 3D structure of *At*HPPD complexed 1TFZ with ligand DAS869 was selected as target protein. In the enzyme-inhibitor complex, the Fe(II) coordinated to the three amino acids and the 1,3-diketone moiety of the DAS869 inhibitor. The distances from the oxygen atoms to the Fe(II) were restrained to a range of 1.9-2.4 Å. This ensured the octahedral geometry and provided a strong ligand orientation and binding force (Yang et al., 2004). Based on the results of the above literature, the native ligand was redocked into corresponding binding pocket. Figure 8 showed that re-docked ligand and natural ligand share the same binding site, whose RMSD values of 0.55 were calculated, confirming the accuracy and feasibility of the docking method of CDOCKER. These 1917 hits obtained through pharmacophore-based screenings were submitted to molecular docking studies by DS v3.5. The distance from Fe(II) to two oxygen atoms of nine candidate ligand was the range of 1.9-2.4 Å. The selection was further obtained 9 compounds (Table 2) based on their -CDOCKER energy, which picked out 2, 4, 1, and 2 molecules from Maybridge, Chembridge, ChemDiv and Specs, respectively.

**Table 2:** The 2D structure of the potential HPPD Inhibitors and the evaluation value.

**Compound ID**	**Structure**	**Fitvalue**	**Estimate**	**-CDOCKER energy**	**p*K*_a_**
Control 1 (mesotrione)	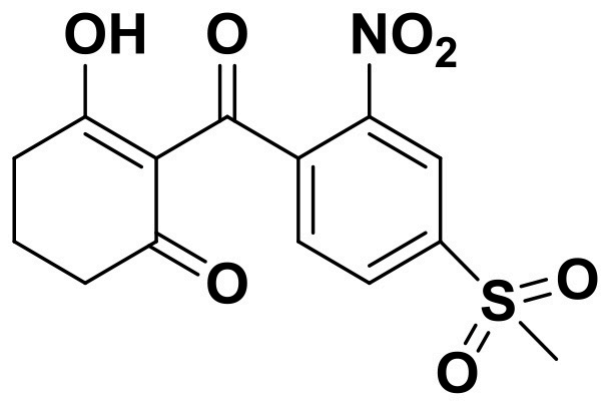	–	–	36.95	2.6 (0.5)
Control 2 (2-(Aryloxyacetyl) cyclohexane-1,3-diones)	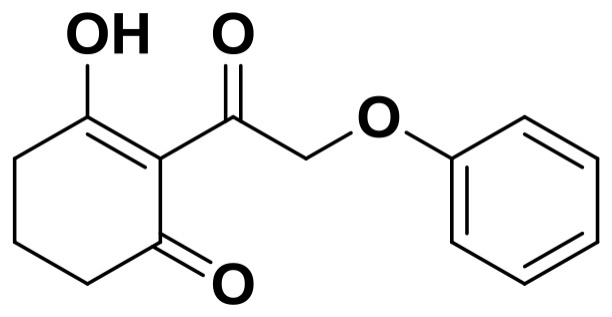	–	–	33.29	4.6 (0.8)
3885 (Chembridge)	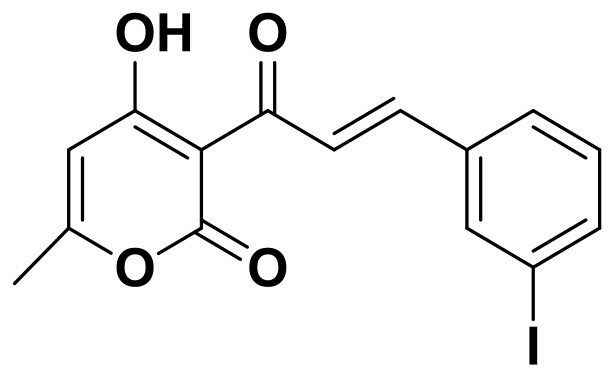	8.31	0.15	32.45	5.1 (0.8)
3881 (Chembridge)	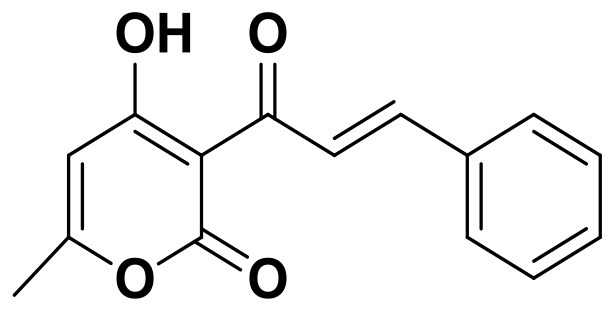	7.06	1.24	29.04	5.1 (0.8)
3882 (Chembridge)	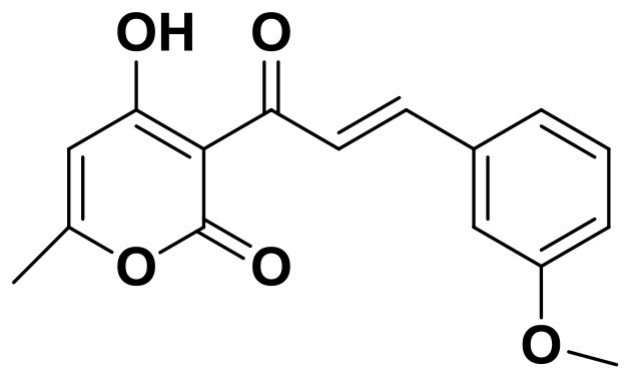	7.17	2.00	28.08	5.1 (0.8)
4293 (Chembridge)	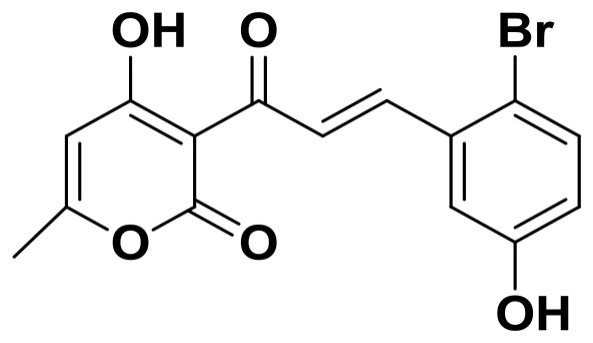	6.87	4.01	28.01	5.2 (0.8)
520 (Maybridge)	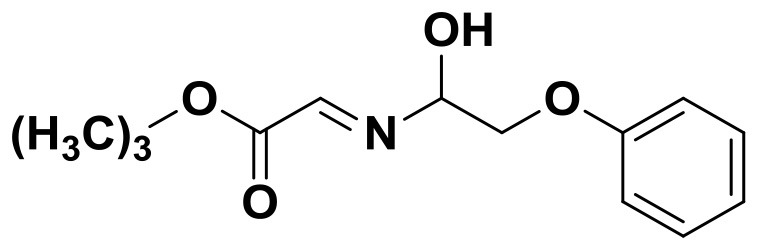	6.73	3.68	29.65	5.8 (0.5)
522 (Maybridge)	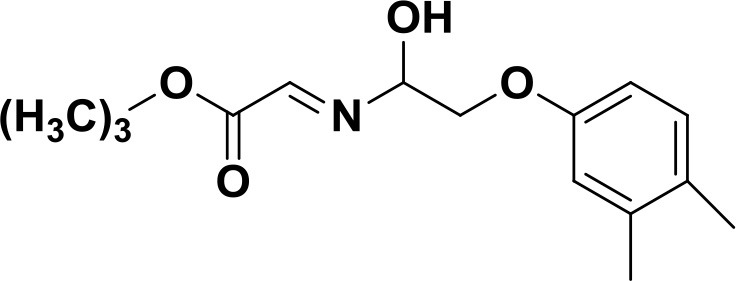	6.78	3.73	28.74	5.8 (0.5)
4798 (ChemDiv)	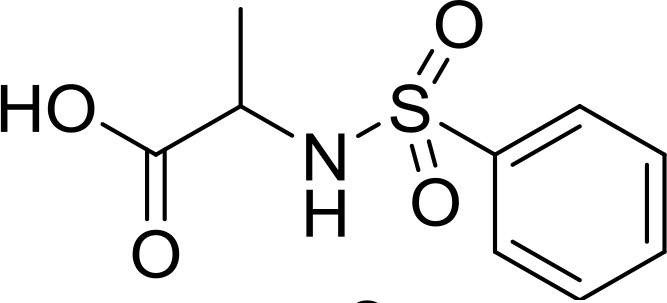	7.23	2.17	30.15	7.8 (0.6)
118 (Specs)	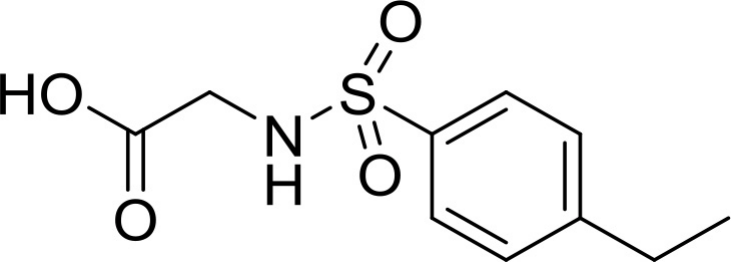	7.26	2.38	29.89	7.8 (0.6)
120 (Specs)	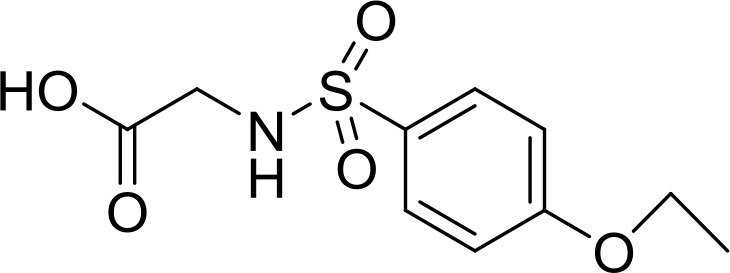	7.26	2.35	29.79	7.8 (0.6)

Compared with the binding affinity the known inhibitors, the selected hits were ranked according to the interaction of the amino acid residues at the binding cavity. Most of the sorted molecules from training set and test set showed good interactions with the critical residues like phe360 and phe403. According to this binding modes and binding affinity, four hit compounds (Table 2) from Chembridge were selected as the target inhibitors. The four compounds selected were structurally similar to the triketones and bind to *At*HPPD in the same pose as the triketone. Compared with the training set of compounds, all potent HPPD inhibitors (compound 1–10) containing 2-benzoylethen-1-ol moiety showed better bioactivity, but the activity without the above structure was reduced, such as compound 11–16 (Figure 2) as well as the test set. The common chemical structure of representative HPPD inhibitors (Figure S1) also contained the 2-benzoylethen-1-ol moiety, which could coordinate to the Fe(II) and interact with phe360 and phe403, as the essential pharmacophore for a significant inhibition of the HPPD activity. The four hit compounds included common subunit of triketone. Two oxygen atoms of hit compounds were responsible for coordinating with Fe(II). It is an effective way to obtain new HPPD inhibitors by lengthening the aryl moiety of triketone compounds according to the literatures (Wang et al., 2016). Aryl in the extended side chain which inserted a C-O between the triketone and side chains formed a more favorable sandwich π-π interaction with residues Phe360 and Phe403 of *At*HPPD in the active pocket. Similarly, a C=C was inserted between the triketones and the aryl moiety of the four hit compounds. In addition, an extensive review on the triketone inhibitors indicated that modification of substituent on the benzoyl was a common way to increase inhibitory activity, but transformation of 3-hydroxycyclohex-2-en-1-one was hardly seen. The 3-hydroxycyclohex-2-en-1-one part was replaced by 4-hydroxy-6-methyl-2H-pyran-2-one of the obtained four compounds and formed favorable π-π stacking interaction with Phe398, which effectively increased the stability of the ligand binding to *At*HPPD. It was also first reported the interaction with Phe398. The predicted p*K*_a_ for the obtained compounds were less than 6.0, and the weak acidity was favorable for plant uptake and conduction.

**Figure 9:**
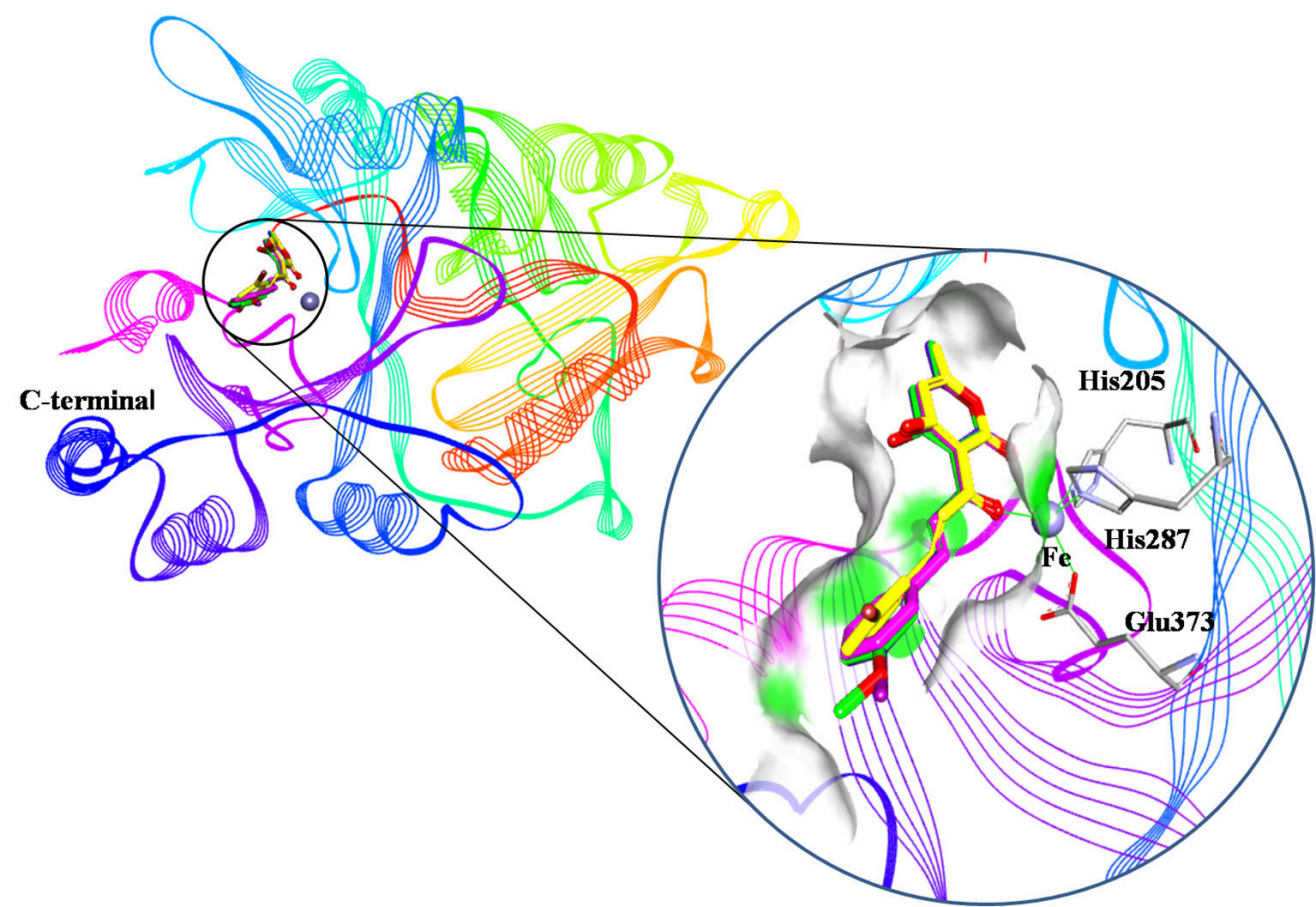
Binding modes of compounds at active pocket. Blue, green, yellow, and pink represented compound 3885, compound 3882, compound 4293, and compound 3881, respectively.

**Figure 10:**
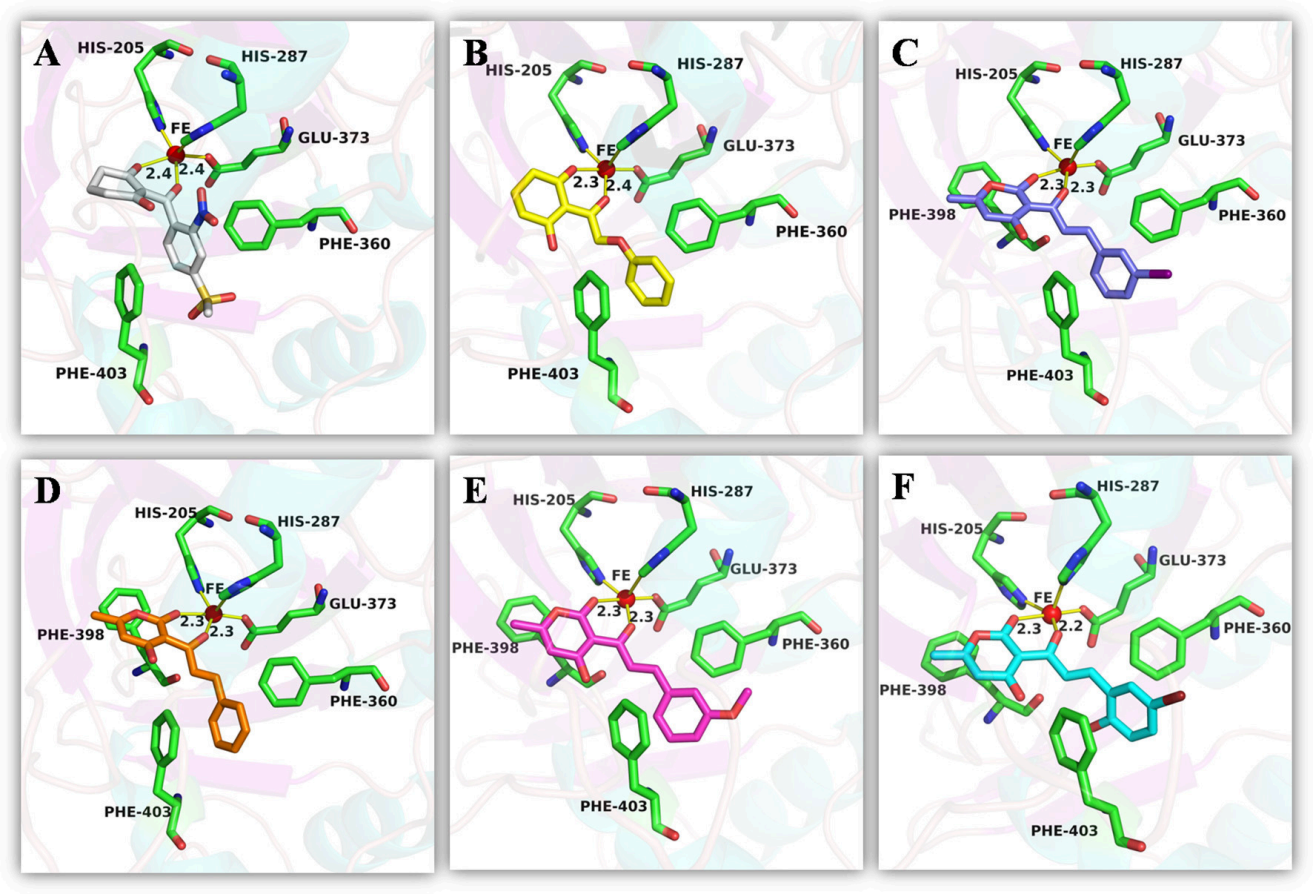
The receptor-ligand interaction of **(A)** control 1 (mesotrione), **(B)** control 2 (2-(Aryloxyacetyl) cyclohexane-1,3-diones), **(C)** compound 3885, **(D)** compound 3881, **(E)** compound 3882, and **(F)** compound 4293 with the HPPD active site.

An analysis of the co-crystallized HPPD-inhibitor complex showed the interaction pattern between the inhibitor and the HPPD binding site (Figure 9). All selected 4 compounds from Chembridge were inserted well into the binding groove and showed metal-coordination binding to Fe(II). The C-terminal harbored a wide cavity exposed to the solvent that accommodated the Fe(II) cofactor. Ferrous ion coordination was fulfilled by the amino acids His205, His287, and Glu373, while the two remaining coordinating water were replaced by the oxygen atoms of inhibitors. When the distances of two oxygen atoms and the Fe(II) were compared (Figure 10), we found that distance from Fe(II) to two oxygen atoms of 4293 was 2.2 and 2.3Å and the distance of Fe(II) with oxygen atoms of compound 3885, compound 3881 and compound 3882 was 2.3 and 2.3Å. However, the distance of control 1 was 2.4 and 2.4Å. For control 2, the distance was 2.3 and 2.4Å. The decreased distances of the Fe(II) from oxygen atoms of four hit compounds may increase HPPD inhibition.

The detailed chemical interactions of the best hits were presented in Figure 10. The benzene ring of the hit compounds sandwiched by the phenyl of Phe360 and Phe403, forming hydrophobic interaction with the two residues. In addition, hit compounds generated another π-π stacking interactions with Phe398, whereas this interaction was disappeared in the binding of control compounds with HPPD. Therefore, the aromatic subunit should be introduced on the 1,3-dione part for design novel HPPD inhibitors in the future, which lead the π-π interaction with phe398 to increasing the binding affinity. All the interactions indicated that the selected compounds were potential HPPD inhibition.

### MD simulations analysis

**Figure 11:**
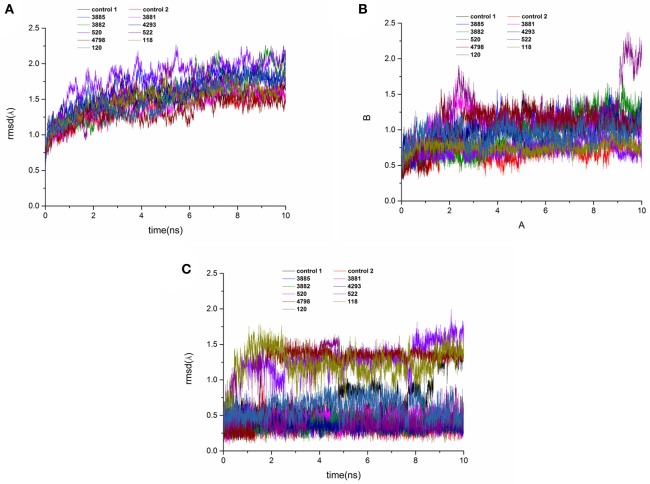
RMSD of the backbone Cα atoms **(A)**, protein active pocket with residues of 5 Å around ligand **(B)** and heavy atoms of ligand **(C)** of the HPPD complexes with reference to the first snapshots as a function of time.

MD simulations were carried out to validate the dynamic interactions between ligands and receptors, and the MM/PBSA program was applied to calculate the binding free energy. To assess the dynamics stability of all the complexes during the MD simulations, root-mean-square-deviations (RMSD) was used to monitor entire MD simulation of each complex. As shown in Figure 11A, all the RMSD values of backbone atoms of protein was very smooth in the whole simulation process maintaining at around 1.5–2.0 Å. Figure 11B showed that the RMSD of the active site of compound 522 suffered a conformational change during the MD simulation after 9 ns. It could be seen that the averaged RMSD of all the heavy atoms of the ligand reached equilibrium at about last 1 ns, which indicated that the all the trajectories was stable during the course of simulation after 1 ns course of simulation in Figure 11C. Therefore, the last 1 ns trajectory was used to analyze the binding free energy and free energy decomposition of all complexes.

For each system, 200 snapshots of each complex were extracted from the last 1 ns stable MD trajectory and used to calculate binding free energy calculations and the results were shown in Table 3. The calculated Δ*G*_bind_ of the four compounds from Chembridge was higher 30 kcal/mol, however, binding free energy of other compounds from Maybridge, ChemDiv and Specs was lower than control and other compounds in Chembridge. The compound 3885 bound systems remained highest than other bound systems. The Δ*E*_vdw_, Δ*E*_ele_, and Δ*G*_SA_ energy were the favorable contribution to Δ*G*_bind_, and the positive free energy (Δ*G*_PB/GB_) displayed adverse effect for all the systems. The Δ*E*_ele_ made the greatest contribution to the binding free energy for all complexes. And electrostatic energy of control 2 and control 1 was lower than compounds from databases. Therefore, the electrostatic interaction should be increased in modification of the compound to strengthen the interactions between each other.

**Table 3:** Contributions of various energy components to the binding free energy (kcal/mol) for the studied inhibitor with HPPD^a^.

**System**	**Δ*E*_vdw_**	**Δ*E*_ele_**	**Δ*G*_PB/GB_**	**Δ*G*_SA_**	**Δ*E*_gas_**	**Δ*G*_sol_**	**Δ*G*_bind_**
Control 1	−36.97 (2.63)	−84.88 (7.04)	91.38 (6.34)	−3.64 (0.07)	−121.85 (6.88)	87.74 (6.30)	−34.11 (5.78)
Control 2	−25.91 (2.90)	−88.39 (5.95)	75.94 (8.06)	−2.96 (0.11)	−114.30 (5.44)	72.99 (7.99)	−41.31 (5.78)
3885	−29.46 (3.02)	−75.59 (5.36)	64.58 (3.82)	−3.48 (0.29)	−105.05 (4.98)	61.10 (2.42)	−43.95 (6.45)
3881	−28.79 (3.16)	−65.68 (5.22)	59.52 (2.69)	−3.72 (0.49)	−94.47 (4.79)	55.8 (2.42)	−38.67 (5.21)
3882	−29.85 (2.94)	−72.88 (5.27)	65.70 (5.21)	−3.35 (0.08)	−102.74 (5.75)	62.35 (5.20)	−40.38 (6.03)
4293	−27.49 (2.24)	−69.23 (4.62)	68.72 (5.91)	−3.57 (0.15)	−96.72 (5.40)	65.15 (5.20)	−31.57 (5.06)
520	−22.72 (3.93)	−130.15 (14.18)	128.30 (12.86)	−3.57 (0.08)	−152.87 (11.46)	124.73 (12.82)	−28.14 (5.32)
522	−29.76 (3.22)	−106.79 (4.36)	110.47 (3.66)	−3.94 (0.09)	−136.55 (4.24)	106.53 (3.62)	−30.02 (4.15)
4798	−21.34 (3.62)	−109.01 (6.46)	117.02 (5.24)	−3.04 (0.05)	−130.35 (3.90)	113.98 (5.21)	−16.37 (4.22)
118	−22.87 (2.06)	−69.24 (4.73)	74.90 (4.30)	−2.70 (0.07)	−92.11 (4.57)	72.20 (4.27)	−19.91 (2.88)
120	−22.03 (2.06)	−81.04 (6.49)	99.98 (5.97)	−3.17 (0.07)	−103.07 (5.94)	96.81 (5.93)	−6.26 (2.17)

a *ΔE_vdw_, van der Waals energy; ΔE_ele_, electrostatic energy; ΔG_PB/GB_, polar solvation energy with the PB model; ΔG_SA_, nonpolar solvation energy with the PB model; ΔE_gas_ = ΔE_vdw_ + ΔE_ele_; ΔG_sol_ = ΔG_PB/GB_ + ΔG_SA_; ΔG_bind_ = ΔE_vdw_ + ΔE_ele_ + ΔG_PB/GB_ + ΔG_SA_*.

The Δ*G*_PB/GB_ of the control 1 (91.38. kcal/mol) was higher than that of the control 2 (75.94 kcal/mol) and compound 3881 (59.52 kcal/mol). The probable causes were that the lengthening the aryl side chain of control 2 and compound 3881 reduced the polar solvation energy. It was found that compound 3881(−28.79 kcal/mol) displayed relative lower Δ*E*_vdw_ energy than those of control 2 (−25.91 kcal/mol). It should be noted that the sum of the nonpolar term (Δ*E*_vdw_ + Δ*G*_SA_ = −32.51 kcal/mol) of 3881 were obviously stronger than that of control 2 with −28.87 kcal/mol. Therefore, these results indicated that nonpolar interactions played a crucial role in the increased binding affinities of 3881 system.

**Figure 12:**
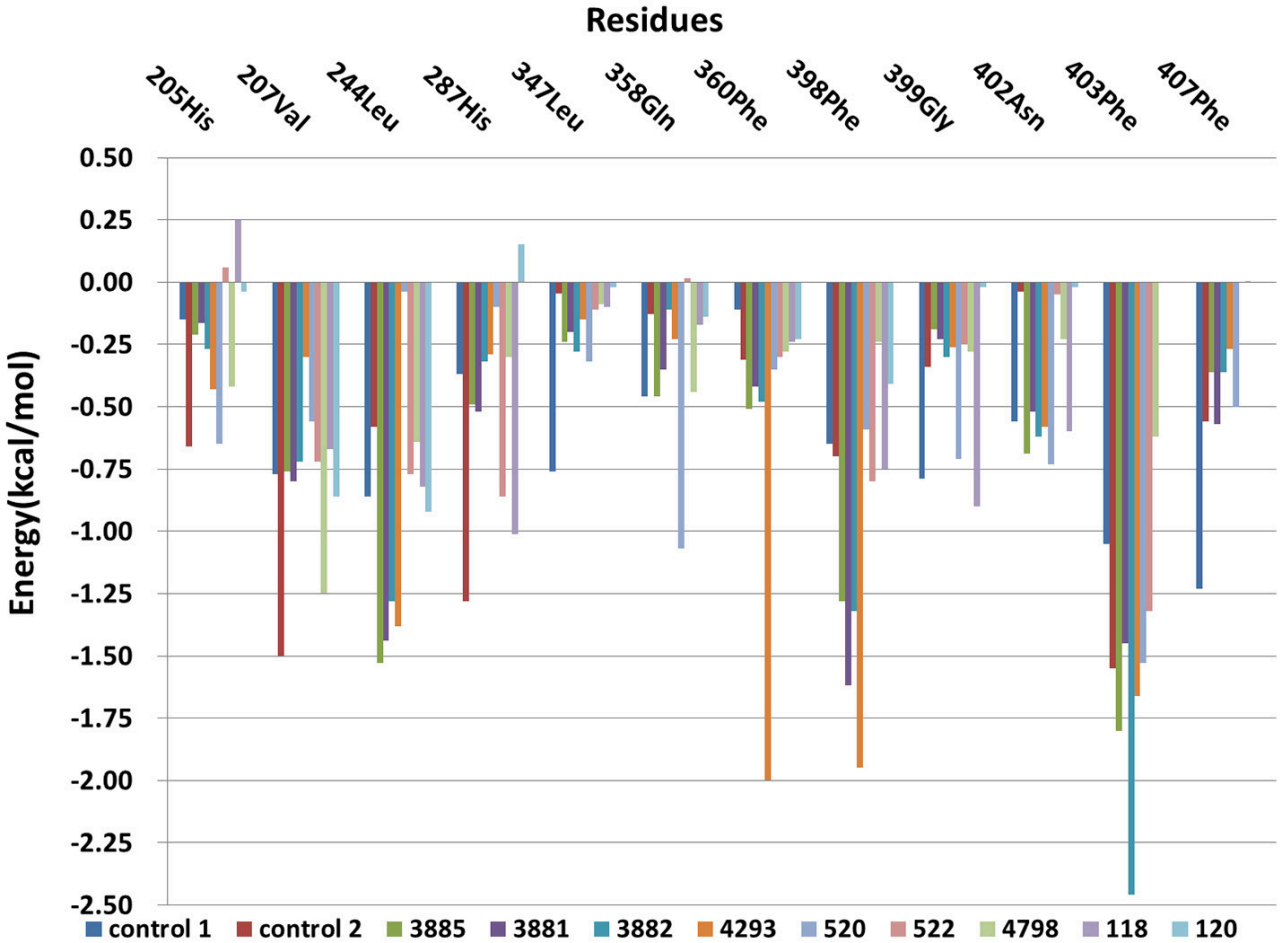
The Per-residue contributions of HPPD to ligand combination.

The free energy decomposition was calculated to investigate the contribution of key residue for the binding process from the last 1 ns stable MD trajectory and the per-residue contribution for the binding of all systems was plotted in Figure 12. Residues Val207, His287, Phe360, and Phe403 made the greatest contribution to binding energy for control 2. And Residues Phe403 and Phe407 was important composition for binding energy of control 1. His205 was unfavorable for the binding energy of compound 118 and His287 had a negative effect on the binding free energy of compound 120. Residues Leu244, Phe398, and Phe403 had a more than 1 kcal /mol free energy contribution to the binding of compound 3881, 3882, 3885, and 4293 and phe403 made the biggest contribution to the binding between compound 3882 and protein with −2.46 kcal/mol. It can be seen that the residues Phe403 and Phe360 has a greater interaction with control 2, four compounds from Chembridge and two compounds from Maybridge than control 1 and other compounds, and it was found that these compounds included extended side chain of aryl that formed a more favorable sandwich π-π interaction. Furthermore, the contribution of residues Phe398 to the binding of four compounds from Chembridge has an obvious increase, indicating the importance of π-π interaction between Phe398 and candidate ligand 3881, 3882, 3885, and 4293. This made a conclusion that molecular dynamics result verified the interaction with Phe398, Phe403, and Phe360 in molecular docking, which may lead to an increase in hydrophobic interaction. During MD simulation, it is regrettable that hydrogen bond could not be found and these findings are consistent with the previous studies (Moran, 2014) that no hydrogen bonds or ionic interactions contributed to the complex.

### Bioactivity analysis

**Figure 13:**
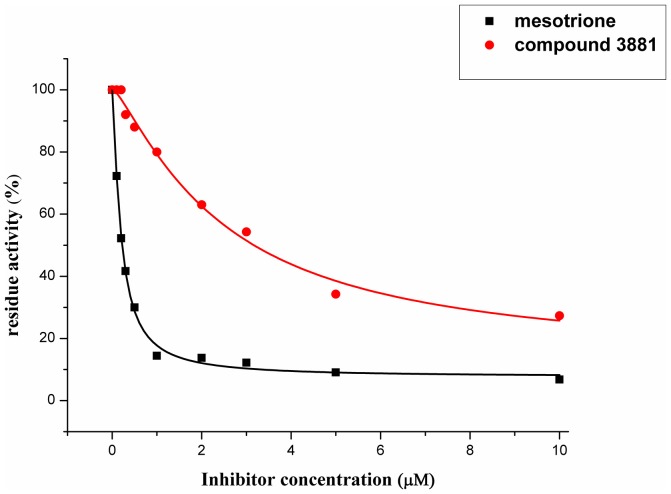
Dose-dependent inhibitory curves of the mesotrione and compound 3881 against *At*HPPD.

Compound 3881, identified *in silico* as promising *At*HPPD inhibitor, was submitted to *in vitro* assays in order to confirm the biological activity. The commercial mesotrione was used as positive control. Figure 13 showed dose-dependent inhibitory activity and the IC_50_ values were calculated. The IC_50_ of mesotrione was 0.204 μM. The inhibition of compound 3881 at 5 μM was 65.89%, and IC_50_ was 2.489 μM, showing good inhibitory activity against *At*HPPD *in vitro* indicating that it may serve as a good template for the design of potent HPPD inhibitor. Furthermore, the activity of compound 3881 was better than compound 10 (IC_50_:11.2 μM) that was parent skeleton of mesotrione in the Figure 2. Compound 3881 was the parent skeleton of all the four hit compounds and exhibited inhibition to *At*HPPD, according to analogous to the similarity property principle (i.e., similar chemical structures share similar biological activities) (Johnson and Maggiora, 1990), so it was inferred that the virtual screening derivative 3882 with iodine, 3885 with methoxyl, and 4293 with bromine and hydroxyl also may show good activity of inhibitor. Therefore, compound 3881 will be used as a template compound for structural modification in the subsequent work to obtain higher activity HPPD inhibitors.

## Conclusions

In summary, a reasonable and hierarchical virtual screening workflow was successfully constructed to identify potential HPPD inhibitors. Specially, 3D-pharmacophore and molecular docking were applied to obtain 4 hits from 111560 compounds. Compound 3881(3-cinnamoyl-4-hydroxy-6-methyl-2H-pyran-2-one) subjected to biological validation against *At*HPPD, showing good inhibitory activity with IC_50_ being 2.489 μM. Molecular docking result indicted that hit compounds generated the bidentate coordination with Fe(II) and formed the sandwiched π-π interaction of the benzene ring with Phe403 and Phe360. In addition, it is firstly reported π-π stacking interactions with Phe398. Therefore, the aromatic subunit should be introduced on the 1,3-dione section for design novel HPPD to improve the binding affinity. The MD simulation and MM/PBSA calculations confirmed interaction with Phe398 to make great contributions to the binding free energy, which suggested that the constructed model was reliable and viable. As far as known, these compounds have not been previously reported as HPPD inhibitors. Therefore, this result offered interesting templates for design of novel and more potent HPPD inhibitors.

## Author contributions

YF and FY: constructed the workflow; Y-NS and K-HY: built the pharmacophore-based screening work; M-QL and H-FC: performed and analysis the molecular docking; J-ZL: developed the MD experiment; Y-NS: carried out the bioassay of HPPD; YF and FY: discussed and concluded the results; YF: completed the paper.

### Conflict of interest statement

The authors declare that the research was conducted in the absence of any commercial or financial relationships that could be construed as a potential conflict of interest.

## Footnotes

^1^SYBYL Version 6.9. Tripos Inc., St. Louis, MO.

^2^Catalyst Version 4.10. Accelrys Inc., San Diego, CA, USA, 2005.

## Supplementary material

Figure S1 Structures of the most representative triketone HPPD inhibitors.

## References

[B1] Ahrens H. Lange G. Muller T. Rosinger C. Willms L. van-Almsick A. (2013). 4-Hydroxyphenylpyruvate dioxygenase inhibitors in combination with safeners: solutions for modern and sustainable agriculture. *Angew. Chem. Int. Ed*. Engl. 52, 9388–9398. 10.1002/anie.201302365 23893910

[B2] Beaudegnies R. Edmunds A. J. Fraser T. E. Hall R. G. Hawkes T. R. Mitchell G. (2009). Herbicidal 4-hydroxyphenylpyruvate dioxygenase inhibitors—a review of the triketone chemistry story from a Syngenta perspective. Bioorg. Med. Chem. 17, 4134–4152. 10.1016/j.bmc.2009.03.015 19349184

[B3] Borowski T. Bassan A. Siegbahn P. E. (2004). 4-Hydroxyphenylpyruvate dioxygenase: a hybrid density functional study of the catalytic reaction mechanism. Biochemistry 43, 12331–12342. 10.1021/bi049503y 15379572

[B4] Brownlee J. M. Johnson-Winters K. Harrison D. H. Moran G. R. (2004). Structure of the ferrous form of (4-hydroxyphenyl) pyruvate dioxygenase from streptomyces avermitilis in complex with the therapeutic herbicide, NTBC. Biochemistry 43, 6370–6377. 10.1021/bi049317s 15157070

[B5] Case D. A. Cerutti D. S. Cheatham T. E. Darden T. A. Duke R. E. Giese T. J. (2017). AMBER 2017. San Francisco, CA: University of California.

[B6] Cho J. E. Kim J. T. Kim E. Ko Y. K. Kang N. S. (2013) The structure-based three-dimensional pharmacophore models for *Arabidopsis thaliana* HPPD inhibitors as herbicide. B. Korean Chem. Soc. 34, 2909–2914. 10.5012/bkcs.2013.34.10.2909

[B7] Dayan F. E. Singh N. McCurdy C. R. Godfrey C. A. Larsen L. Weavers R. T.. (2009) β-Triketone inhibitors of plant *p*-hydroxyphenylpyruvate dioxygenase: modeling comparative molecular field analysis of their interactions. J. Agric. Food Chem. 57, 5194–5200. 10.1021/jf9005593 19435355

[B8] Darden T. York D. Pedersen L. (1993) Particle mesh Ewald: an *N*-log(N) method for Ewald sums in large systems. J. Chem. Phys. 98, 10089–10092. 10.1063/1.464397

[B9] Debnath A. K. (2002). Pharmacophore mapping of a series of 2,4-diamino-5-deazapteridine inhibitors of *Mycobacterium avium* complex dihydrofolatereductase. J. Med. Chem. 45, 41–53. 10.1021/jm010360c 11754578

[B10] Essmann U. Perera L. Berkowitz M. L. Darden T. Lee H. Pedersen L. G. (1995). A smooth particle mesh Ewald method. J. Chem. Phys. 103, 8577–8593. 10.1063/1.470117

[B11] Frisch M. J. Schlegel H. B. Scuseria G. E. Robb M. A. Cheeseman J. R. Montgomery J. A. (2004). Gaussian 03. Wallingford, CT: Gaussian Inc.

[B12] Gogoi D. Baruah V. J. Chaliha A. K. Kakoti B. B. Sarma D. Buragohain A. K. (2016). 3D pharmacophore-based virtual screening, docking and density functional theory approach towards the discovery of novel human epidermal growth factor receptor-2 (HER2). J. Theor. Biol. 411, 68–80. 10.1016/j.jtbi.2016.09.016 27693363

[B13] Green J. M. (2014). Current state of herbicides in herbicide-resistant crops. Pest Manag. Sci. 70, 1351–1357. 10.1002/ps.3727 24446395

[B14] Guedes R. A. Serra P. Salvador J. A. Guedes R. C. (2016). Computational approaches for the discovery of human proteasome inhibitors: an overview. Molecules 21, 1–27. 10.3390/molecules21070927 27438821 PMC6274525

[B15] Hornak V. Abel R. Okur A. Strockbine B. Roitberg A. Simmerling C. (2006). Comparison of multiple amber force fields and development of improved protein backbone parameters. Proteins 65, 712–725. 10.1002/prot.21123 16981200 PMC4805110

[B16] Huang M. Yang D. Y. Shang Z. C. Zou J. W. Yu Q. (2002). 3D-QSAR studies on 4-hydroxyphenylpyruvate dioxygenase inhibitors by comparative molecular field analysis (CoMFA). Bioorg. Med. Chem. Lett. 12, 2271–2275. 10.1016/S0960-894X(02)00432-8 12161114

[B17] Jakalian A. Jack D. B. Bayly C. I. (2002). Fast, efficient generation of high-quality atomic charges. AM1-BCC model: II. Parameterization and validation. J. Comput. Chem. 23, 1623–1641. 10.1002/jcc.10128 12395429

[B18] Johnson M. Maggiora G. M. (1990). Concepts and applications of molecular similarity. Am. Math. Mon. 12, 96–97.

[B19] Kovaleva E. G. Lipscomb J. D. (2008). Versatility of biological non-heme Fe(II) centers in oxygen activation reactions. Nat. Chem. Biol. 4, 186–193. 10.1038/nchembio.71 18277980 PMC2720164

[B20] Lee D. L. Prisbylla M. P. Cromartie T. H. Dagarin D. P. Howard S. W. Provan W. M. (1997). The discovery and structural requirements of inhibitors of p-hydroxyphenylpyruvate dioxygenase. Weed Sci. 45, 601–609.

[B21] Lei K. Hua X. W. Tao Y. Y. Liu Y. Liu N. Ma Y.. (2016). Discovery of (2-benzoylethen-1-ol)-containing 1,2-benzothiazine derivatives as novel 4-hydroxyphenylpyruvate dioxygenase (HPPD) inhibiting-based herbicide lead compounds. Bioorgan. Med. Chem. 24, 92–103. 10.1016/j.bmc.2015.11.032 26682702

[B22] Li P. F. Merz K. M. (2014). Taking into account the Ion-induced dipole interaction in the nonbonded model of Ions. J. Chem. Theory Comput. 10, 289–297. 10.1021/ct400751u 24659926 PMC3960013

[B23] Lin J. F. Sheih Y. L. Chang T. C. Chang N. Y. Chang C. W. Shen C. P.. (2013). The interactions in the carboxyl terminus of human 4-hydroxyphenylpyruvate dioxygenase are critical to mediate the conformation of the final helix and the tail to shield the active site for catalysis. PLoS ONE 8:e69733. 10.1371/journal.pone.0069733 23950902 PMC3739788

[B24] Meazzaa G. Scheffler B. E. Tellezb M. R. Rimando A. M. Romagni J. G. Duke S. O. (2002). The inhibitory activity of natural products on plant p-hydroxyphenylpyruvate dioxygenase. Phytochemistry 60, 281–288. 10.1016/S0031-9422(02)00121-8 12031447

[B25] Mitchell G. Bartlett D. W. Fraser T. E. Hawkes T. R. Holt D. C. Townson J. K.. (2001). Mesotrione: a new selective herbicide for use in maize. Pest Manag. Sci. 57, 120–128. 10.1002/1526-4998(200102)57:2<120::AID-PS254>3.0.CO;2-E 11455642

[B26] Moran G. R. (2014). 4-Hydroxyphenylpyruvate dioxygenase and hydroxymandelate synthase: exemplars of the α-keto acid dependent oxygenases. Arch. Biochem. Biophys. 544, 58–68. 10.1016/j.abb.2013.10.022 24211436

[B27] Moran R. G. (2005). 4-Hydroxyphenylpyruvate dioxygenase. Arch. Biochem. Biophys. 433, 117–128. 10.1016/j.abb.2004.08.015 15581571

[B28] Ndikuryayo F. Moosavi B. Yang W. C. Yang G. F. (2017). 4-Hydroxyphenylpyruvate dioxygenase inhibitors: fromchemical biology to agrochemicals. J. Agric. Food Chem. 65, 8523–8537. 10.1021/acs.jafc.7b03851 28903556

[B29] Neidig M. L. Decker A. Choroba O. W. Huang F. Kavana M. Moran G. R.. (2006). Spectroscopic and electronic structure studies of aromatic electrophilic attack and hydrogen-atom abstraction by non-heme iron enzymes. Proc. Natl. Acad. Sci. U.S.A. 103, 12966–12973. 10.1073/pnas.0605067103 16920789 PMC1559736

[B30] Peters M. B. Yang Y. Wang B. Fusti-Molnar L. Weaver M. N. Merz K. M. (2010). Structural survey of zinc-containing proteins and development of the Zinc AMBER Force Field (ZAFF). J. Chem. Theory Comput. 6, 2935–2947. 10.1021/ct1002626 20856692 PMC2941202

[B31] Purpero V. M. Moran G. R. (2006). Catalytic, noncatalytic, and inhibitory phenomena: kinetic analysis of (4-hydroxyphenyl) pyruvate dioxygenase from *Arabidopsis thaliana*. Biochemistry 45, 6044–6055. 10.1021/bi052409c 16681377

[B32] Raspail C. Graindorge M. Moreau Y. Crouzy S. Lefebvre B. Robin A. Y. (2011). 4-Hydroxyphenylpyruvate dioxygenase catalysis identification catalytic residues and production of a hydroxylated intermediate shared with a structurally unrelated enzyme. J. Biol. Chem. 29, 26061–26070. 10.1074/jbc.M111.227595 PMC3138293 21613226

[B33] Ryckaert J. P. Ciccotti G. Berendsen H. J. C. (1977). Numerical integration of the Cartesian equations of motion of a system with constraints: molecular dynamics of n-alkanes. J. Comput. Phys. 23, 327–341. 10.1016/0021-9991(77)90098-5

[B34] Sakkiah S. Thangapandian S. John S. Kwon Y. J. Lee K. W. (2010). 3D QSAR pharmacophore based virtual screening and molecular docking for identification of potential HSP90 inhibitors. Eur. J. Med. Chem. 45, 2132–2140. 10.1016/j.ejmech.2010.01.016 20206418

[B35] Siehl D. L. Tao Y. M. Albert H. Dong Y. X. Heckert M. Madrigal A.. (2014). Broad 4-hydroxyphenylpyruvate dioxygenase inhibitor herbicide tolerance in soybean with an optimized enzyme and expression cassette. Plant Physiol. 166, 1162–1176. 10.1104/pp.114.247205 25192697 PMC4226376

[B36] Silva T. C. Pires M. D. S. de Castro A. A. da Cunha E. F. Caetano M. S. Ramalho T. C. (2015). Molecular insight into the inhibition mechanism of plant and rat 4-hydroxyphenylpyruvate dioxygenase by molecular docking and DFT calculations. Med. Chem. Res. 24, 3958–3971. 10.1007/s00044-015-1436-3

[B37] Sitkoff D. Sharp K. A. Honig B. (1994). Accurate calculation of hydration free energies using macroscopic solvent models. J. Phys. Chem. 98, 1978–1988. 10.1021/j100058a043

[B38] Sutton P. Richards C. Buren L. Glasgow L. (2002). Activity of mesotrione on resistant weeds in maize. Pest Manag. Sci. 58, 981–984. 10.1002/ps.554 12233193

[B39] Vishnoi S. Agrawal V. Kasana V. K. (2009). Synthesis and structure-activity relationships of substituted cinnamic acids and amide analogues: a new class of herbicides. J. Agric. Food Chem. 57, 3261–3265. 10.1021/jf8034385 19368353

[B40] Wang D. W. Lin H. Y. Cao R. J. Ming Z. Z. Chen T. Hao G. F.. (2015). Design, synthesis and herbicidal activity of novel quinazoline-2,4-diones as 4-hydroxyphenylpyruvate dioxygenase inhibitors. Pest Manage. Sci. 71, 1122–1113. 10.1002/ps.3894 25185782

[B41] Wang D. W. Lin H. Y. He B. Wu F. X. Chen T. Chen Q.. (2016). An efficient one-pot synthesis of 2-(aryloxyacetyl) cyclohexane-1,3-diones as herbicidal 4-hydroxyphenylpyruvate dioxygenase inhibitors. J. Agric. Food Chem. 64, 8986–8993. 10.1021/acs.jafc.6b04110 27933872

[B42] Wang J. M. Wolf R. M. Caldwell J. W. Kollman P. A. Case D. A. (2004). Development and testing of a general amber force field. J. Comput. Chem. 25, 1157–1174. 10.1002/jcc.20145 15116359

[B43] Witschel M. (2009). Design, synthesis and herbicidal activity of new iron chelating motifs for HPPD-inhibitors. Bioorg. Med. Chem. 17, 4221–4229. 10.1016/j.bmc.2008.11.006 19028100

[B44] Woodyard A. J. Hugie J. A. Riechers D. E. (2009). Interactions of mesotrione and atrazine in two weed species with different mechanisms for atrazine resistance. Weed Sci. 57, 369–378. 10.1614/WS-08-175.1

[B45] Wu C. S. Huang J. L. Sun Y. S. Yang D. Y. (2002). Mode of action of 4-hydroxyphenylpyruvate dioxygenase inhibition by triketone-type inhibitors. J. Med. Chem. 45, 2222–2228. 10.1021/jm010568y 12014960

[B46] Xu Y. L. Lin H. Y. Cao R. J. Ming Z. Z. Yang W. C. Yang G. F. (2014). Pyrazolone–quinazolone hybrids: a novel class of human 4-hydroxyphenylpyruvate dioxygenase inhibitors. Bioorgan. Med. Chem. 22, 5194–5211. 10.1016/j.bmc.2014.08.011 25182962

[B47] Yang C. Pflugrath J. W. Camper D. L. Foster M. L. Pernich D. J. Walsh T. A. (2004). Structural basis for herbicidal inhibitor selectivity revealed by comparison of crystal structures of plant and mammalian 4-hydroxyphenylpyruvate dioxygenases. Biochemistry 43, 10414–10423. 10.1021/bi049323o 15301540

[B48] Yang Y. Shen Y. L. Liu H. X. Yao X. J. (2011). Molecular dynamics simulation and free energy calculation studies of the binding mechanism of allosteric inhibitors with p38a MAP kinase. J. Chem. Inf. Model. 51, 3235–3246. 10.1021/ci200159g 22097958

