# Supplementary figures and images for: Combination of Virtual Screening Protocol by in Silico toward the Discovery of Novel 4-Hydroxyphenylpyruvate Dioxygenase Inhibitors
Source: Front Chem. 2018 Feb 6;6:14. doi: 10.3389/fchem.2018.00014 (PMC5807903; doi:10.3389/fchem.2018.00014)

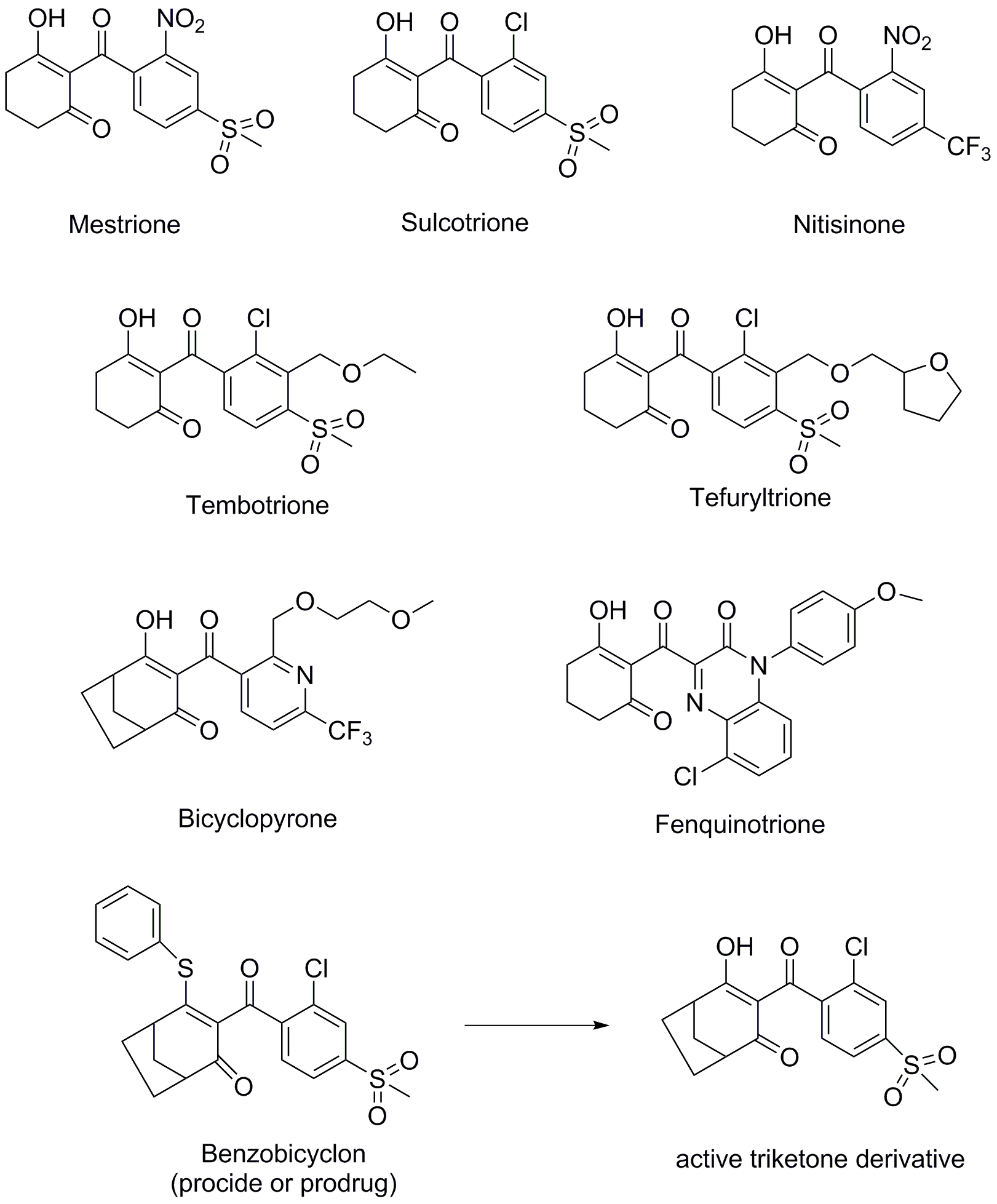

Supplement: Figure S1 — Structures of the most representative triketone HPPD inhibitors. [file Image1.TIF]
